# Automated disassembly of e-waste—requirements on modeling of processes and product states

**DOI:** 10.3389/frobt.2024.1303279

**Published:** 2024-03-22

**Authors:** José Saenz, Torsten Felsch, Christoph Walter, Tim König, Olaf Poenicke, Eric Bayrhammer, Mathias Vorbröcker, Dirk Berndt, Norbert Elkmann, Julia Arlinghaus

**Affiliations:** ^1^ Department of Robotic Systems Department, Fraunhofer IFF, Magdeburg, Germany; ^2^ Department of Industrial Metrology and Digital Assistance Systems, Fraunhofer IFF, Magdeburg, Germany; ^3^ Department of Energy Systems and Infrastructures, Fraunhofer IFF, Magdeburg, Germany; ^4^ Fraunhofer IFF, Magdeburg, Germany

**Keywords:** robotics, recycling, digital twin, sustainability, robotic applications for sustainability, de-manufacturing, industry 4.0

## Abstract

Automated disassembly is increasingly in focus for Recycling, Re-use, and Remanufacturing (Re-X) activities. Trends in digitalization, in particular digital twin (DT) technologies and the digital product passport, as well as recently proposed European legislation such as the Net Zero and the Critical materials Acts will accelerate digitalization of product documentation and factory processes. In this contribution we look beyond these activities by discussing digital information for stakeholders at the Re-X segment of the value-chain. Furthermore, we present an approach to automated product disassembly based on different levels of available product information. The challenges for automated disassembly and the subsequent requirements on modeling of disassembly processes and product states for electronic waste are examined. The authors use a top-down (e.g., review of existing standards and process definitions) methodology to define an initial data model for disassembly processes. An additional bottom-up approach, whereby 5 exemplary electronics products were manually disassembled, was employed to analyze the efficacy of the initial data model and to offer improvements. This paper reports on our suggested informal data models for automatic electronics disassembly and the associated robotic skills.

## 1 Introduction

Current megatrends, from climate and demographic change to the tense geopolitical situation, are increasing the pressure on manufacturing companies in Germany and Europe. Value chains that have been predominantly based on the use of primary raw materials (principle: new materials for new products) are no longer sustainable for ethical, economic and political reasons. In a world with finite availability of raw materials and energy, new ideas are needed to achieve the ambitious global climate and environmental goals.

Business and politics have already recognized the need to establish a circular economy as a response to these challenges. In the circular economy, materials and products are reprocessed by so-called Re-X processes (e.g., recycling, remanufacturing, etc.) so that they can be reused. Thus, raw materials can be returned to the product cycle on a large scale through recycling or products through remanufacturing. Such approaches address challenges due to rising energy prices and dependence on raw materials including so-called Critical Raw Materials. Since 1996, the Recycling Management Act (KrWG, 2023), which came into force in Germany, has focused on minimizing the adverse effects of waste generation and treatment on the environment. Further laws and draft laws at the European level increase the requirements for the dismantlability, reparability and traceability of products of all kinds. These include, among others, the EU Eco-Design Directive (Europäische Kommission, 2021), the German Supply Chain Sourcing Obligations Act. (bmas, 2023). and the upcoming Critical Raw Materials Act (European Commission, 2023). These laws not only respond to the general public opinion against practices such as “planned obsolescence”, but also try to bring various challenges regarding energy, climate and environmental protection in line with a stable economy.

Currently, all manufacturing industries are affected by the challenges of the circular economy, from aviation and automotive to consumer goods. Production is very resource-intensive, especially with regard to the availability of the necessary raw materials. Recycling electrical and electronic equipment can provide direct access to secondary raw materials such as copper, aluminum, steel, gold, silver and rare earths. Currently, 99.8% of metal ores in Germany have to be imported (Holtschneider and Hajek, 2021). Of the approximately 53.6 million tons of waste from electrical and electronic equipment (WEEE) generated annually worldwide, only 17.4% is currently recycled, although this waste contains approximately €50 billion in value from precious metals such as gold, silver, and copper (Forti et al., 2022). For example, one ton of computer mainboards contains as much gold as 45 tons of gold-bearing ore. (Billerbeck, 2022). The situation is similar for cell phones and other high-value electronic goods. Indeed, even though 98% of the gold, copper, silver, palladium and platinum can already be recovered using ordinary recycling processes, the overall low level of products being recycled represents a huge lost potential. The reasons for the relatively low recycling rate vary. It is estimated that eight percent of all old electrical and electronic equipment ends up in residual waste. Another seven to 20% are exported to other countries as e-waste or second hand products (Forti et al., 2022). Currently, the recycling potential of these products, e.g., in the form of recovery of secondary raw materials or reuse, is not being exploited.

In the future, the dismantling of old electrical equipment to access their raw materials will be an important component of the circular economy. To counteract the current shortage of skilled workers and ensure that the processes are economically-viable, we believe that the dismantling of used electrical equipment should to be primarily executed through automation. A prerequisite for the automation of these previously manually performed tasks is the availability of uniform data structures for storing, exchanging and processing relevant information about the electrical equipment and the associated dismantling processes. This will make it easier to generate dismantling processes for new products in the future and to respond to product changes, while reducing the engineering efforts and costs traditionally associated with such flexible automation.

The main contribution of this paper is to formulate a vision and methodology for how flexible robots of the future could be used to automate disassembly tasks for Re-X applications, to derive from this vision clear requirements on that system, in particular with respect to the role of data and the associated modeling of product and process data specific to the disassembly tasks.

This paper is structured as follows. We begin with an overview on the state of the art for automated disassembly, and formulate a generic model for the individual tasks involved. We then propose a workflow for supporting an automated, robot-based disassembly of various products, including suggestions for dealing with varying levels of *a-priori* information about the product to be disassembled. We then derive requirements on a data model to support the envisioned workflow, and identify the main elements of the data model and connections. To test the usability and completeness of the proposed data model, we then manually disassembled 5 exemplary electronic waste products (desktop computers) to extract the mainboard from the housing. We report on the process, highlighting modifications to the data model and also commenting on specific issues related to availability of *a-priori* information, the quality of this information, and its relation to the proposed informal data models. We finish with an outlook for next steps towards our vision for automated disassembly of products for Re-X activities and in support of a circular economy.

## 2 Materials and methods

As with many robotics applications, the disassembly of WEEE for Re-X activities such as recycling or remanufacturing is highly interdisciplinary and builds on many different domains of knowledge. In this section, we will formulate our initial vision for how robots can be used for flexible disassembly tasks. Then we will investigate the state of the art for the specific disciplines and topics involved, highlighting gaps with respect to our proposed application. In particular, we will look at the overall state of the art for automated disassembly in general, before looking into more detail at the topics of digital models for Re-X processes, descriptions of disassembly processes, and then, one step deeper, at the topic of robot skills. Following the state of the art, we will describe challenges and requirements on automated disassembly processes in greater detail, and present the results of our top-down approach of using existing standards from a variety of different areas to create the informal data models necessary to support our methodology for robot-based disassembly processes. In the final sub-section, we will define the test scenario we used as a bottom-up methodology to validate the informal data models formulated through the top-down approach.

### 2.1 Proposed workflow for automated disassembly tasks

**FIGURE 1:**
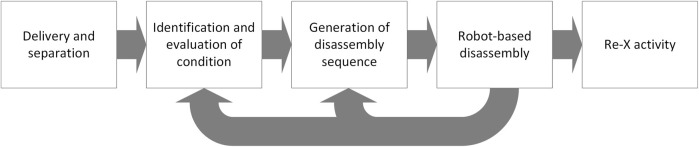
Flowchart of automated disassembly process, starting with delivery of product to be recycled and ending with individual Re-X activity adapted from (Kopacek and Kopacek, 2003).

In our evaluation, we begin with the collection of WEEE and their delivery to a specific recycling center. There they are separated into separate fractions, typically according to the 6 categories defined by (Parlament, 2018). Once in a specific fraction, the individual products need to be identified and their condition needs to be evaluated. The condition of the product is particularly important, as missing or damaged sub-components will influence the next step of generating a suitable sequence of tasks to disassemble the product. This step is highly dependent both on the evaluated condition and on the available information about the product. In a perfect world, full product information will be available, enabling an automated system to access CAD and other product data to determine the proper actions for non-destructive disassembly. Using the information from the part identification (e.g., vision-guided) and acting according to the disassembly sequence, the robot-based disassembly will be able to generate motions and actions for the robots, eliminating the current need to fully program all robot paths. The steps of part identification, condition evaluation, generation of disassembly sequence and robot-based disassembly will then be iteratively executed until the desired level of disassembly has been achieved and the individual sub-components are at a level of purity that allows for efficient Re-X processes. Figure 1 highlights these main processes.

An interesting characteristic of this workflow is that it is highly data driven. Either the automated disassembly system collects the data by itself during the phases of identification and condition evaluation, or it draws upon data from various available sources. In either case, the semantic mapping of the product and its subcomponents to the formulation of a disassembly sequence that can then be understood and executed by a robot places high demands on the data modeling, Given the scope of WEEE, in particular the range of manufacturers, the varieties of products, and the current heterogenous landscape with respect to data modeling, traditional methods of robot engineering whereby all individual movements are programmed, is not a feasible approach. Furthermore, from a data perspective, the need for clear standards to ensure interoperability becomes more relevant.

### 2.2 State of the art

Conventional methods of recycling electrical goods include mechanical, thermal and chemical processes to enable further recovery of certain materials. For example, pollutants are first removed manually before mechanical shredding can be performed. Then, valuable materials (e.g., ferrous and non-ferrous metals, plastics and minerals) are extracted through various processes. If old equipment is not shredded, then the disassembly is primarily executed manually (Überblick, 2020). However, manual disassembly is typically less attractive due to its relatively high cost and increasing shortage of labor.

In principle, it is important for efficient recycling that used products are broken down into individual parts by disassembly and ideally into different material fractions. The better individual materials are separated from one another, the higher the value of their further usability as secondary raw materials and the lower the energy input for the respective recycling process. Accordingly, it is advantageous to separate individual materials as non-destructively as possible. By automating disassembly processes, there is the potential for significantly more products to actually be disassembled and thus for materially separated fractions to be fed into recycling processes. Developed technical capabilities for automated disassembly are still relevant for remanufacturing processes, although here it is even more important to make disassembly as damage- and destruction-free as possible.

In the following sub-sections, we will focus on the topic of automated disassembly for Re-X processes before taking a deeper look at the associated data models available. Then we will look at available models for describing disassembly processes before taking a final look at the topic of robotic skills. In this way we progress from a high-level view of complete disassembly systems, down to the ability of robots to operate in a flexible, data-driven manner through skills-based methodologies.

#### 2.2.1 Automated disassembly for Re-X processes

There are already some large international companies active in the field of disassembly of products for recycling and remanufacturing. For example, Apple Inc. has an iPhone recycling facility “Daisy” (Staff, 2020) developed to automatically remove and recycle electronic components from old iPhone models. These recovered components and materials are in turn incorporated into new devices.

Renault has in 2021 the Refactor factory (Reuters, 2021) in Nils, France to enable recycling and remanufacturing of old vehicles. Here, only certain Renault models are in focus and the disassembly tasks are performed manually. Some joint ventures aiming at closing loops in the automotive sector are also known (e.g., Audi with Remondis and Encory as a joint venture of BMW with Alba Group and now part of Interzero). For certain automotive components, independent remanufacturers have also established themselves on the market (e.g., Borg Automotive).

In the aircraft sector, remanufacturing for actuators and turbines is already widespread. As in the automotive sector, dismantling activities are mainly manual. The activities are based on a known data situation, since contract-based remanufacturers (such as MTU Maintenance) are mainly used here and there are high requirements for documentation and verification obligations in the aviation sector.

In Germany, the collection and dismantling of electrical and electronic equipment takes place at the municipal level. There are over 2400 collection points, e.g., in the form of recycling centers, hazardous waste collection vehicles or depot collection containers. (Forti et al., 2022). In some cases, initial treatment takes place here in the form of sorting or manual dismantling. After an initial material separation, the components are either transported directly or through intermediaries to specialized recycling companies (e.g., for copper recycling) or are also recycled at waste incineration plants (e.g., for plastic parts).

These initiatives all have in common that they are very specialized on a small product group. They require a high level of personnel effort for setup, commissioning and operation and are also characterized by low flexibility when introducing new products. As mentioned in Section 2.1, the authors believe that in order to raise the share of Re-X processes and to establish an efficient circular economy, it is essential to combine Industry 4.0 methods (e.g., with respect to data modeling) with advanced automation techniques to flexibly execute disassembly processes.

#### 2.2.2 Digital models for Re-X processes

Building on the idea that digital models of products will be key to future automated disassembly operations for WEEE, it is useful to understand the range of product information and models currently available. Indeed, there are a number of industry-led and regulatory initiatives that tie into this topic and we will briefly highlight these and identify how they can be used for the proposed vision. The authors firmly believe that considering the complexity of the task (i.e., digital models for all electronics products to support Re-X processes), the most feasible approach is to use existing international standards and initiatives and focus on how these can be used or minimally adapted to support the needs arising from automated disassembly. Indeed, given the current interest in the field from both consumers and companies, as well as the current regulatory focus, the authors see that there are at the least opportunities to connect with existing activities in a meaningful way.

Using the European WEEE Legislation (EUROPÄISCHE, 2012) as a starting point, we see that there is an initial distinction between complete and finished equipment and subcomponents. These subcomponents are considered unfinished products with no direct function for the end-user, as they typically require further processing, assembly, or installation in order to be used by a customer. This means there is a general categorization of EEE products into one of the 6 current categories, based on their size, their type, and the possible content of hazardous substances. These are: temperature exchange equipment (e.g., refrigerators, etc.), screens, monitors and screens larger than 100 cm^2^, lamps, large equipment (with any external dimension larger than 50 cm), small equipment (all external dimensions are smaller than 50 cm), and small IT and telecommunication equipment.

Moving beyond the WEEE legislation, there are a number of taxonomies from industry and research that focus on easing ease of data exchange for purchasing and sourcing purposes. Among these are the ECLASS, ETIM, proficl@as, and the UNSPSC. In the case of automated disassembly, it is important to be able to classify both products as well as their constituting components. ECLASS for example, focuses on the classification of products and is based on ISO-compliant characteristics according to ISO 13584:2010–12 Industrial automation systems and integration - Parts library - Part 42: Descriptive methodology: Methodology for structuring families of parts. It also applies the standard IEC 61360–2:2012 Standard data element types with associated classification scheme for electrical components—Part 2: EXPRESS dictionary scheme. There are currently approximately 46 product classes defined within ECLASS, sorted in a tree structure with 4 levels. According to this system, products can be described with over 28,000 individual characteristics including manufacturer name, product type, weight, and dimensions.

While it is currently in an early stage of conception, the European initiative for a Digital Product Passport (DPP) is very noteworthy. The DPP is specifically designed for consumers, companies, and authorities, to support shopping decisions and to simplify repair and recycling activities (Adisorn et al., 2021). There are still many open considerations with regard to details such as where the data is to be stored (in a central database or locally), how to deal with the inevitable complexity of various data types and how to ensure that the data is up to date (Götz et al., 2022).

A further consideration is the question of data management. The Industrie-4.0 concept of the Asset Administration Shell (AAS), while also in an evolving state, is gaining traction in industry and offers interoperability for the types of data that we foresee as being necessary (Plattform industrie 4.0, 2022).

In order to merge product data for the preparation of disassembly, different approaches for the use of digital models or DTs are described in the literature. The first approaches to using digital models for recycling and remanufacturing exist (Wang and Wang, 2019). The need to increasingly develop and establish DTs in the context of products and their life cycles is also described, e.g., to support product service systems (PSS) (Zhang et al., 2019). PSS are used to offer new services based on the DT. Thereby, information from the twin is provided and at the same time captured data from the use phase as well as identification, finding and disassembly processes are consistently fed back (Kritzinger et al., 2018; Massonet et al., 2020). DTs on the product level can accordingly provide important information for the disassembly of the respective product and its use in the life cycle. A detailed overview of currently used methods in the field of remanufacturing can be found in (Rizova et al., 2020).

The AAS (Plattform industrie 4.0, 2022) can be used for the technological implementation of DTs. It is a joint concept of the “Plattform Industrie 4.0″and industry associations for the realization of the DT. It is intended to become the standardized basis to create the future, open and decentralized ecosystems and to realize innovative applications and business models (Verwaltungsschale, 2022). The subject of the current standardization efforts are so-called sub-models of the management shell. The partial models are used to formalize which data should be available in which form for different application domains, for example, for simulation. This is one focus of the work in the project for the domain automated disassembly.

Currently, more than 450 different IoT platforms are available (i-SCOOP, 2021). However, only a part of them works with the concept of DTs in different forms, for concrete use cases. A uniform, standardized technological implementation, for example, by using the management shell, is often not given (Christian et al., 2021). One of the best-known Industrie-4.0 frameworks is BaSys 4.0 together with the reference implementation Eclipse BaSyx. Based on this, an ecosystem of methods and applications was developed with partners, which supports the operation of DTs in the form of management shells, thus enabling highly flexible value networks and making their use easily accessible to industry (Bayrhammer et al., 2022).

With a view towards the concept of a digital disassembly twin, the authors seek to build on these current results and will continue to advance them with new ideas in the application case of disassembly.

#### 2.2.3 Description of disassembly processes

In our vision for a data-driven, automated disassembly of WEEE in Section 2.1, we also highlighted the need for a disassembly sequence, which is description of the processes involved. For the development of disassembly sequences, a variety of Disassembly Sequence Planning (DSP) methods have been described in the literature. A recent overview of different methods, trends and research needs is provided by (Zhou et al., 2019; Guo et al., 2020). Often, DSP is divided into three steps: 1) the determination of a disassembly mode, 2) the disassembly modeling, and 3) subsequent planning methods. The disassembly mode determines whether a complete or partial disassembly, aimed only at the recovery of high-value components, is to take place. Basically, DSP methods create the disassembly sequences based on existing knowledge about the individual components. Priority relationships are determined by analyzing CAD models. This process is performed manually according to the state of the art (Zhou et al., 2019). Individual methods of DSP incorporate additional criteria, such as remanufacturing economics for components, into sequence planning (Xia et al., 2020).

Existing information on assembly can and should be used in automated disassembly. Descriptions of the processes often exist for the assembly of products. The REFA - Verband für Arbeitsgestaltung (association for work design) provides recommendations for structuring and presenting work processes (Industrial Engineering, 2015). The REFA flow structure is a sequence and linking of flow sections of a defined workflow. Typical forms of the representations of expirations are tabular overviews or visual expiration diagrams in those in each case the row of necessary individual work steps are specified.

Another way to represent assembly sequences are precedence graphs for situation-oriented worker guidance (Wiesbeck, 2014). Assembly instructions for the individual work steps are created to further substantiate the processes. The assembly instructions describe the specific work steps and necessary tools.

For the creation or extension of digital models in the context of disassembly, the inclusion of human experience knowledge (e.g., regarding structured description of the disassembly process, handling, etc.) is also important. Such knowledge is generated in activities and documented by experts.

#### 2.2.4 Robot skills

In the end, it is necessary to be able to convert disassembly sequences (in whatever form they are formally defined) into concrete actions that can be directly executed by the robot-based automation system.

The term “skill” is often used in relation to robot functions and robot programming. However, the approach of past research often reflects different points of view and with slightly different goals. In some cases, it refers to basic functions (services) that are provided for the realization of the actually intended processes (Schlegel et al., 2015). Often the term “skill” is used more in the sense of software engineering (Herrero et al., 2015) and tries to achieve the goals of functional programming in the context of robot programming. In this case, skills are seen as functions that are used in the programming environments and run-time systems of the respective software-frameworks. Here, they act within the architectural constraints or implications of an underlying robot control architecture. We see this as efforts towards the simplification of robot programming by providing both structure - in the sense of the linkage of partial functions, and a certain re-usability of once developed software-modules for use in similar or different applications. The work outlined in (Reiser et al., 2022), for example, was also motivated by the concept of re-use by modularization of sub-tasks using skills, whereby the aspect of co-simulation and virtual commissioning was also addressed.

However, the research on robot skills also tries to address the problem of the reusability of software implementations in the sense of software engineering, but also with respect to variable physical processes. Here, the aspect of appropriate structuring of functions according to an approach of (incomplete and/or imprecise) perception and consequent action generation is added. Human behavior in solving these tasks often serves as a model for the design of skill systems that focus on this aspect. This in turn is also related to advanced forms of programming by demonstration (Pedersen and Krüger, 2015)- Another question is, what set of skills is necessary or useful to be used in a domain such as manufacturing? The result was concrete skills like “pick” and “place” (Pedersen et al., 2016). However, these were quite high-level and relatively abstract, and needed further breakdown into smaller skills.

### 2.3 Challenges on and requirements for automated disassembly

In this section, we will highlight some of the largest challenges associated with automated disassembly and will then formulate some general requirements on any system for automated disassembly. Furthermore, we will look at the workflow proposed in Section 2.1 in greater detail, highlighting processes and associated informal data models that are necessary for the types of automated disassembly processes we envision.

Generally speaking, the disassembly or dismantling of products cannot be compared with a simple reversal of the original production or assembly. In new production, the individual parts and assemblies are in a defined, known and new condition. Disassembly of used goods, on the other hand, is characterized by many unknowns and uncertainties. For example, bolts may be rusted, screw heads may be worn, components may be deformed or damaged, or they may be missing entirely. In addition, challenges arise from glued components or other joint connections that are not designed for automated and non-destructive disassembly (e.g., loosening of clips, flipping of levers, loosening of latches). This underlines the importance of the iterative nature of the disassembly process, with component identification and condition evaluation as the basis for determining the correct disassembly sequence to execute.

Another challenge in the dismantling of products is the quality and availability of data on the product. In the previously identified examples of industrial Re-X processes, the manufacturers are involved in the recycling of their own products. Thus, they have excellent data on their own products and can use it to make decisions (e.g., which modules should be reused, which ones should rather be sent for recycling) and to make the disassembly and associated Re-X processes more efficient. This data includes, for example, parts lists, functional descriptions and assembly instructions. For disassembly, three basic scenarios are currently conceivable.

1) Comprehensive source data: A complete geometry description in the form of a CAD model and (dis)assembly-relevant additional information are available for a product/assembly. The real assembly corresponds to the available data.
2) Incomplete or non-up-to-date initial data: Partial model data are available. However, these are not completely applicable to the products at hand (e.g., due to different product versions or generations). Adjustments or changes made during the life cycle of the product are not included in the model data.
3) Missing initial data: There is no model data available, only information about the domain of the component group.

The availability of data is largely dependent on the type of dismantling and remanufacturing company. In general, a distinction is made between original equipment manufacturers, contract-based remanufacturers and independent remanufacturers (Lange, 2017). Current initiatives such as Gaia-X (Gaia, 2023), Catena-X (Catena-X Automotive Network, 2023) and Manufacturing-X (Initiative, 2023) are already striving to find solutions to the challenges of linking and sharing product data across company borders and can make important contributions here.

While trends in regulation and business practices hint at a future whereby comprehensive data is available, a situation with incomplete or missing initial product data is more likely the case for current disassembly applications. Our approach therefore considers all three situations.

Based on the state of the art, we see that there are a wide variety of standards and data models available, however none that explicitly address the specific tasks associated with disassembly for Re-X processes. In particular, we see the following trends.

• The representation of disassembly processes is not clearly describable with existing systems or standards.
• A more analytical approach to information structuring and task sequence generation will need to be complemented by the description of robot skills
• Robot skills will furthermore be a bridge, connecting the description of manual disassembly processes (through formal representation) and their automatic execution through a robot
• Depending on the availability of *a-priori* product information, different workflows are needed to ensure that the disassembly sequence can be generated and these need to be saved and leveraged to support future disassembly processes

**FIGURE 2:**
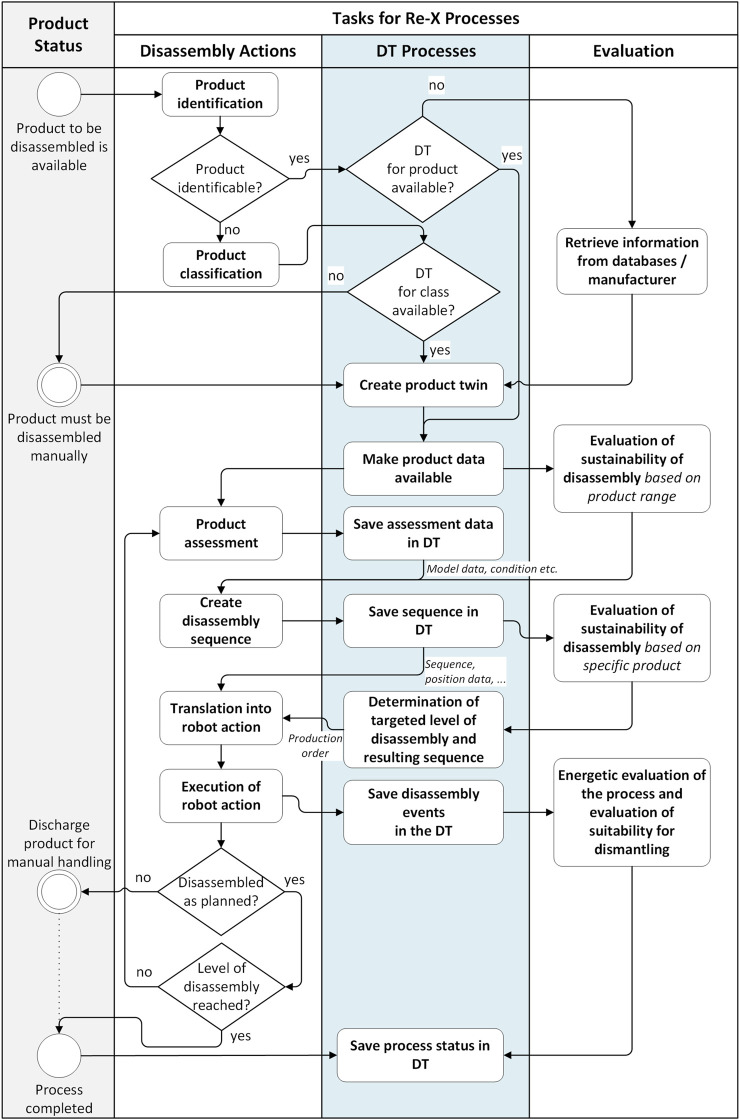
Data connections between the Re-X tasks, including disassembly actions, the associated DT processes, and evaluation/valuation assessment processes.

The informal data models we propose are based on the more detailed processes as described in Figure 2. We foresee three separate swim lanes to differentiate between the physical processes associated with disassembly (adapted from Figure 1), the DT processes, and additional evaluation and valuation assessment processes. As previously mentioned, our process model is designed to handle all three situations with respect to *a-priori* product data. Thus, either the data is available from the manufacturer or from a database (based on previous experience), or it is gathered manually. The evaluation/valuation assessment is designed ensure that the costs associated with disassembly (e.g., robot energy costs, etc.) are in balance with the income that can be generated through the Re-X processes. While out of the scope of this article, innovative business models will be a key component towards ensuring that automated disassembly for Re-X processes will be viable on a large scale. Regarding human-robot collaboration as an aspect for the disassembly processes, we expect that unexpected situations or product conditions can result in a failure to complete the disassembly as originally planned. In this case, the product should be discharged from the robot cell for further manual handling, as highlighted in Figure 2. Due to the inherently hazardous nature of numerous disassembly processes (e.g., shearing, etc.), we do not recommend collaboration as defined in (Behrens et al., 2015), but rather either sequential collaboration or even co-existence. Furthermore, this manual process should also be recorded and transcribed such that an eventual existing DT can be updated, or to support the creation of a new product-specific DT.

### 2.4 Procedure for the selection of the disassembly process

In this section, we build on the generic model from Section 2.3 to propose a more concrete set of informal data models. These individual informal data models will then be described in greater detail in the following sub-sections.

**FIGURE 3:**
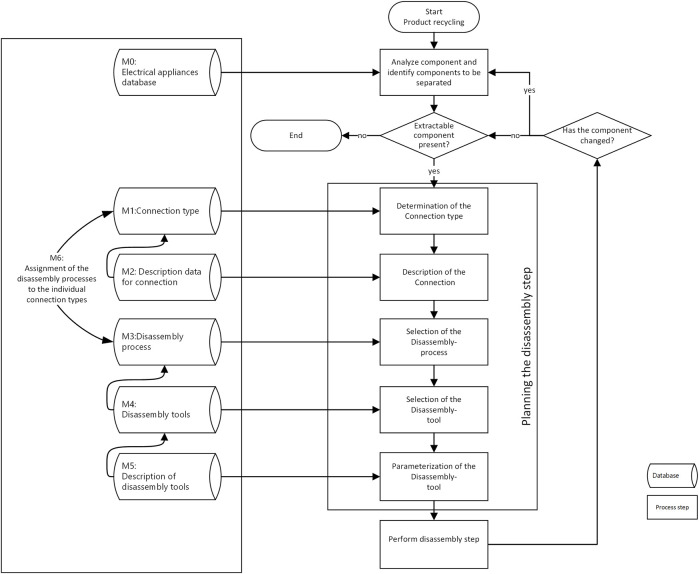
Overview of the complete disassembly processes and the associated informal data models for each step.

As a general overview (Figure 3), we begin with the classification of the product in order to perform the disassembly multiple, iterative steps. The disassembly is then performed until a previously defined disassembly target or level of disassembly (e.g., disassembly of the mainboard from the housing) has been reached. This is determined by a number of factors and is tied to the valuation process and business models.

We have initially defined seven different informal data models (see M0-M7 in Figure 3), which we initially model as a database. The content of these will be described in the following sub-sections.

#### 2.4.1 Initial data modeling

Here we introduce our informal data models and how they relate to each other. In each individual step, a previously defined or recognized component is then separated from the product during disassembly. The sequence of the disassembly steps and thus the selection of the component to be disassembled next in each case can be carried out in different ways.

1) Use of existing information from product documentation (product passport, manuals, CAD models, etc.)
2) Use of existing information from previous disassembly processes of similar products (e.g., documentation of manual disassembly processes)
3) As well as through the use of information (*ad hoc*) from reporting

Once the product has been classified and the disassembly target has been defined, the actual disassembly can then take place.

##### 2.4.1.1 Step 1: product identification and analysis

As a first step, we foresee that the part identification process will likely draw on a database of electrical appliances (see M0 in Figure 3). Here, a product-level description is sufficient and we expect to draw on available standards such as E-CLASS.

The product assessment, i.e., the recording of the respective product type to be dismantled (product classification) as well as the condition of the product (including configurations, signs of aging, etc.), plays a central role in the selection of the dismantling process or tool.

##### 2.4.1.2 Step 2: analysis and description of the connection

During disassembly, the connections between individual parts that were made during the original production process must be taken apart. After selecting or defining the component, the next step is therefore to analyze the type of connection between the component to be separated and the product. In this step, the types of connection in the product are analyzed and described. Connections (in electrical equipment) are typically made by a joining method defined according to DIN 8593. While this standard provided a good starting point, we see that there are also other additional types of connections which are also relevant in electronic products. These can be characterized as combinations of the existing DIN methods and consist of objects such as hinges, hooks.

**FIGURE 4:**
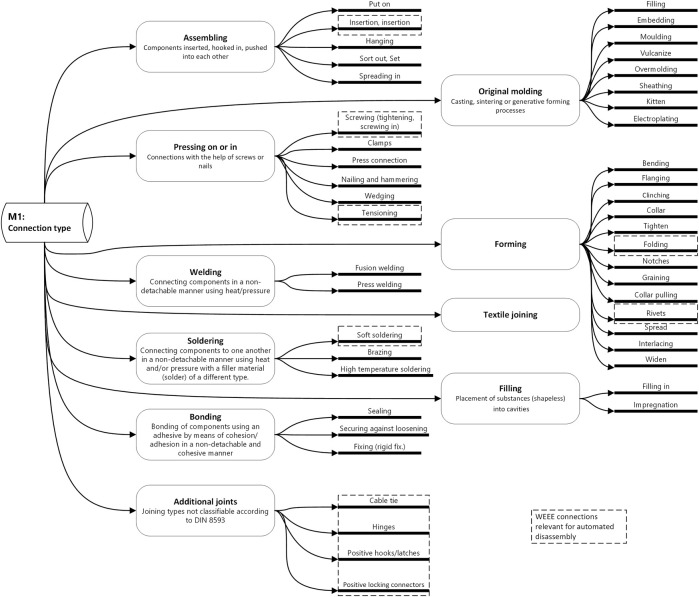
Database on connection types, based on DIN 8593, including overview of those most relevant for WEEE.

Figure 4 shows an overview of the main categories of connections from DIN 8593 (model M1) and their sub-categories. We have highlighted the sub-categories most relevant to WEEE products.

In Table 1 we highlight the connection types we consider most relevant for WEEE disassembly processes. These connection types will then later be used during the benchmarking task of manually disassembling 5 computers.

**TABLE 1:** Overview of connection types and assigned connection groups according to DIN 8593 with additional, specific characteristics.

Connection type	Connection group	Specific characteristic
Insertion	Assembling	Swivel angle, removal direction
Clamping	Pressing on or in	Contact direction and pressure force
Screwing	Pressing on or in	Thread and screw head size
Tensioning	Pressing on or in	Clamping force
Folding	Forming	Material, sheet thickness, number of layers
Soft Soldering	Soldering	Material, melting temperature
Hinge	Additional joints	Opening angle and force, spring-loaded
Hook (positive locking)	Additional joints	Direction and length of pressure
Cable tie	Additional joints	Material, cable bundle diameter
Positive locking connectors	Additional joints	Pulling force, gripping points

After identifying the type of connection in the product survey, the connection should be further described (model M2 from Figure 3) with the aim of supporting disassembly processes. This means that each connection is described in terms of general characteristics (e.g., position, accessibility, etc.) and more specific characteristics such as the thread size for screws or tolerances for pressed-together components. Here typical standards such as ISO 5408:2009 “Screw threads—Vocabulary” are useful to describe details of fasteners. Other details such as the position within the assembly (according to a part-specific coordinate system), forces/moments involved (e.g., the tightening torque of a bolt during production, whether glue was used, or whether a bolt is countered), the reachability (e.g., is the screw hidden behind another part) and the part condition (e.g., rusted) are all important for disassembly. We further believe that a systematic sorting according to the type of joining (e.g., force, form or material closure) will also be useful when selecting the correct disassembly process.

##### 2.4.1.3 Step 3: choice of disassembly process and disassembly tools

Continuing along the processes outlined in Figure 3, we find it advantageous to use the connection type to support the identification of which disassembly process (model M3 from Figure 3) to execute. Going further, we have defined the specific disassembly tools (model M4 from Figure 3) that correspond to the combination of connection type and disassembly process are assigned to each relevant connection type. This is a prerequisite for planning the robot actions in disassembly. When selecting the disassembly processes, one can be guided by the disconnection processes according to DIN 8580 “Manufacturing processes - terms, classification”.

The assignment of several tools to one type of connection is necessary because different tools may have to be used for different states of the same connection. For example, heavily aged or worn screw connections can no longer be loosened by disassembly with a screwdriver, but must be loosened by other separation processes (e.g., cutting, machining or removal). Therefore, there is not a single, logical choice for disassembly tool based solely on the connection, but rather various sources of information need to be collected and analyzed here.

**FIGURE 5:**
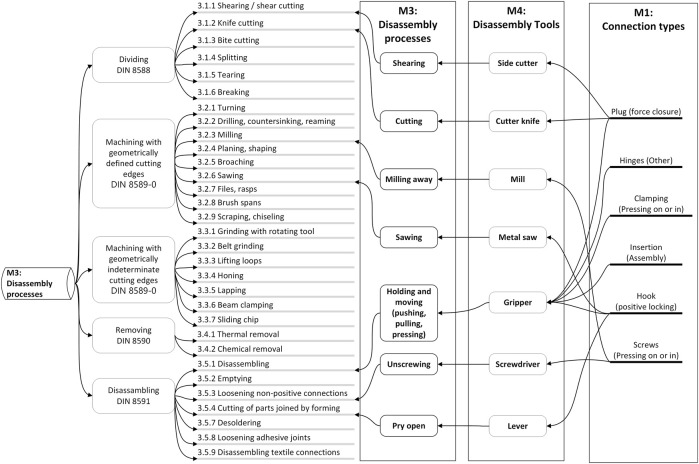
Connections between disassembly processes, disassembly tools and back to connection types.

Continuing along this path, we also see that each disassembly tool therefore also has different characteristics, depending on the specific features of the respective connection (size, material, connection forces, etc.). These are in model M6 from Figure 3. For each tool there is therefore a corresponding parameter set in which the use of the tool is possible (definition of a validity range by parameterization). Figure 5 highlights some exemplary disassembly tools that are associated with the most relevant disassembly processes and connection types.

These models serve to support the engineering processes of specifying the hardware of the overall system, ranging from the robot, the tooling and grippers, and any peripheral actuators and sensors. These models will basically inform the hardware requirements. Our approach can be described as the following: define initial models to support specification of hardware (e.g., robot, tools, peripheral components), implement pilot system based on initial requirements and for specific set of products (e.g., focus only on tower personal computers, vs. all types of WEEE), review system performance with increasing product variance and update all models and automation hardware as needed. The evaluation tasks (see Figure 3) will in turn support decision-making with regard to identifying trade-offs related to robotic system hardware (e.g., which tooling to include, which products to focus on) and to support eventual updates to the system in terms of product types to be processed and corresponding system hardware. In summary, this hardware perspective is focused on the desire to benefit from modularity and composability of just the right robotic components in order to come up with an optimized and cost-effective solution.

In addition to this hardware-centric view, there are also software considerations concerning the required flexibility for disassembling different products in various conditions at run-time. While it would be generally beneficial to have a strictly standardized hardware, challenges such as a desire to allow for hardware evolution and to avoid vendor lock-in make some kind of hardware abstraction necessary. This requires standardized interfaces with documented and comparable performance levels as well as detailed models of both hardware and embedded software behavior. We believe that the use of DTs for modelling and AAS for a standardized implementation will be a strong enabler to achieve these goals.

In Table 2 we extracted the most relevant disassembly processes for WEEE from DIN 8580 and define corresponding disassembly tools. We later use this exact list of processes as part of our informal data model when we manually disassemble end-of-life computers to extract their mainboards.

**TABLE 2:** Overview of Disassembly Processes we propose and their correspondence to DIN 8580.

Disassembly process	Disassembly tool	Corresponding manufacturing process according to DIN 8580
Shearing	Side cutter	Shearing (Dividing)
Cutting	Cutter knife	Knife cutting (Dividing)
Milling	Mill	Milling (Machining)
Sawing	Metal saw	Sawing (Machining)
Holding (and Moving)	Gripper	Disassembling (Disassembling)
Unscrewing	Screwdriver	Loosening non-positive connections (Disassembling)
Pry open	Lever	Cutting of parts joint by forming (Disassembling)

As previously stated, our explicit goal with the creation and use of these informal data models is to support the automatic execution of robot disassembly tasks, without requiring traditional robot programming methods. Therefore, we foresee that the robot will be able to select and plan the use of the tools according to the features detected in the product assessment. The associated tool parameters, such as the necessary degrees of freedom, the associated process forces (e.g., cutting forces) and their ability to be combined with other tools and skills, are all important to allow for the parameterization of robot skills.

#### 2.4.2 Process execution

The ultimate goal of the work presented here is to be able to execute the generated disassembly sequences automatically. The formal disassembly sequence does not yet represent a program that can be executed on a robot or a machine in general. Also, some information is still missing. Other information is too fuzzy and must be supplemented. In some cases, this can only take place at run-time. Finally, assignments to concrete resources such as stations, robots, and tools are not given. We want to solve these problems with a skills-based approach, which is specifically tailored to the needs of disassembly processes. In this way, we enable the automatic execution of the disassembly sequences without any further engineering effort like constructing a specific solution as well as setting up and programming that system. Regarding the implementation of the automation system, we assume that a suitable basis for hyper-flexible automatic disassembly can be provided. This should be possible by using a suitable design of connected workstations, which are equipped with flexible robots, sensors as well as a wide range of tools. Please note, that this work does not address either the concrete specification and choice of tools nor planning problems such as resource allocation across multiple workstations or any optimization of utilization. Instead, we focus on enabling the execution of a disassembly sequence for a broad range of instances of a given product to be disassembled on the available generic equipment.

We use the term “skill” here in the sense of powerful software functions that can each perform a sub-task of the disassembly process while maintaining a high degree of flexibility as well as a high degree of self-containment. Flexibility means that the functions tolerate changing environmental boundary conditions and object states. It does not mean that the software must be able to run with arbitrary robots or other components. Self-containment means that, given a manageable set of pre- and post-conditions regarding the state of the product being disassembled, there is powerful decision making and error handling contained within the skill implementation. Skills should be able to be concatenated, where the sequence is in principle given by the disassembly description and ideally requires little adaptation or addition in the transition from one skill to the next.

**FIGURE 6:**
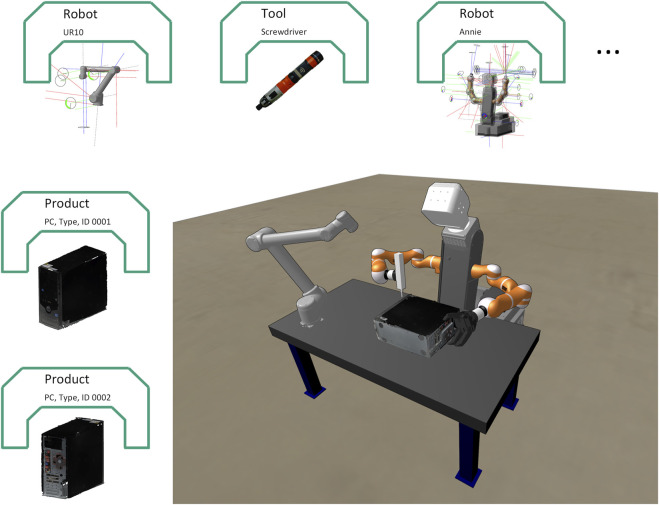
Model of a test set-up of a disassembly station using DTs for asset representation.

Our concept of skills provides for two variations. One variant includes implementations of actions based on analytical planning methods. A second variant are AI-based skills, which are primarily used when a high level of dexterity is required for the execution of subtasks, which cannot be modeled with conventional motion primitives. The analytical approach typically has the problem that generated motion often cannot be executed directly because the models normally used represent the robot type only and are too imprecise in the sense that they do not accurately describe the real-world instances. This is not only true for the problem of recognizing and locating objects to be handled, but also for accurate models of the robots and tools used. The authors of (Garg et al., 2021) attest to the need for more precise models for offline programming and demonstrate the use of a DT. We also rely on the use of DTs as the basis of analytical skills, but in addition propose the use of the Industry 4.0 ecosystem, in which corresponding sub-models of the AAS (Plattform industrie 4.0, 2022) can be defined and used in order to describe all model information that is required for autonomous planning. In our system, the online version of the AAS makes consistent and up to date model data available to all software components that offer services for analytical planning. Figure 6 shows the mapping of products and resources in the form of AASs.

In our approach, a skill is first and foremost a software artifact. A skill may nevertheless still involve dependencies to specific hardware. From a software architectural point of view, anything that can be invoked within a sequence of actions (see section 3.2) could be considered to be a skill. One of the most trivial implementations would be a block of conventionally programmed movements. Such a skill would be quite simple, both with respect to its implementation and its capability. Thus, it may be an appropriate means to solve a specific problem for a specific task in a specific situation, but it would not be reusable in any meaningful way. Reusability is a major goal of robot software modularization and is a priority in our development of robot skills. We consider the creation of a skill-based robot program to be an end-user programming activity, i.e., a task carried out by domain experts instead of software engineers. This is also true in the case of automatically generated skills-based programs or sequences. In our view, the end-user should not be tasked with implementing new skills from scratch. Rather, we believe they should be able to implicitly spawn instances of pre-built skills by putting them in sequence, providing parameters and/or perform any required setup procedures. In order to enable this, we have limited the scope of applications to the disassembly processes as discussed in the previous sections. Another design goal of these pre-built skills is to reduce the complexity when implementing the disassembly operation for a particular device. This relies on the consequent use of autonomous behavior within each skill, which in turn is based on availability of suitable models in the form of DTs. Here, the run-time use of model information provided by DTs is mandatory. If a skill changes the state of any asset within the workspace, it is also required to update corresponding DTs after execution or incrementally during execution.

In summary, it can be said that a suitable skill system is potentially capable of translating the disassembly process descriptions we have proposed into concrete robot actions. Integration into the Industry 4.0 conceptual model with the concepts of the DT and the AAS is a suitable means of providing the necessary model data not only of the products to be disassembled, but also of the operating resources, especially the robots, sensors and tools.

### 2.5 Benchmarking and evaluation

The general applicability of the methodology and informal data models was verified in an initial test based on the manual dismantling of specific WEEE. In our benchmarking scenario, five different computers (towers) were disassembled manually and all steps were documented. For all computers, the pre-selected level of disassembly was to remove the mainboards from the housing. For this task, we allowed for the use of publicly available, existing information such as from online product documentation. The aim of our tests is on the one hand to determine whether all relevant information can be collected and structured accordingly, and whether the corresponding disassembly processes can be determined according to our informal data models. Specifically, we want to know whether the connection types, disassembly processes, disassembly tools and robot skills we defined from the top-down methodology contained the language and descriptions needed when carrying out the tasks. Therefore, these and future further tests are intended to expand and supplement the methodology with missing connections, processes and tools (extensibility of the methodology and informal data models).

#### 2.5.1 Description of benchmarking scenario

Table 3 shows an overview of the devices used to evaluate the methodology.

**TABLE 3:** Overview of the waste electrical devices used for the benchmarking evaluation.

No.	PC designation	Illustration of the devices (no. 1 to 5 from left to right)
1	Dell Precision T3400 (RS182)	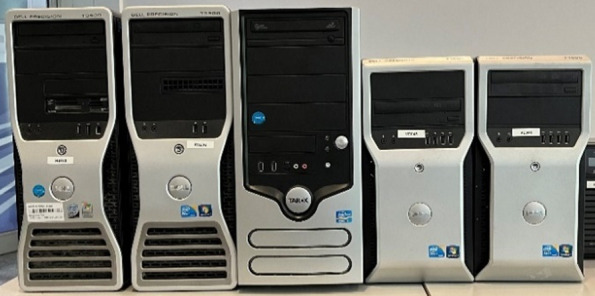
2	Dell Precision T3500 (RS232)	
3	Tarox (RS036)	
4	Dell Precision T1600 (VE045)	
5	Dell Precision T1600 (VE046)	

The benchmarking evaluation was executed as follows. In a first step, a small team of three to four people performed the individual disassembly tasks in the order defined in Figure 2. They were asked to document their steps using terminology from the various informal data models (Figure 3; Figure 5). They were allowed to access the internet during the part identification and use any available documentation to define their own disassembly sequence. The first, high-level tasks as identified from the user manual available online are listed in Table 4.

**TABLE 4:** List of individual steps required to remove the mainboard from the computer housing for PC #5 (Dell Precision T1600) according to online manual.

Step number	Individual task
1	Open and remove side panel of PC housing
2	Open and remove front panel
3	Remove expansion board from mainboard
4	Remove all cable connections to mainboard
5	Unscrew mainboard and remove it from housing

**FIGURE 7:**
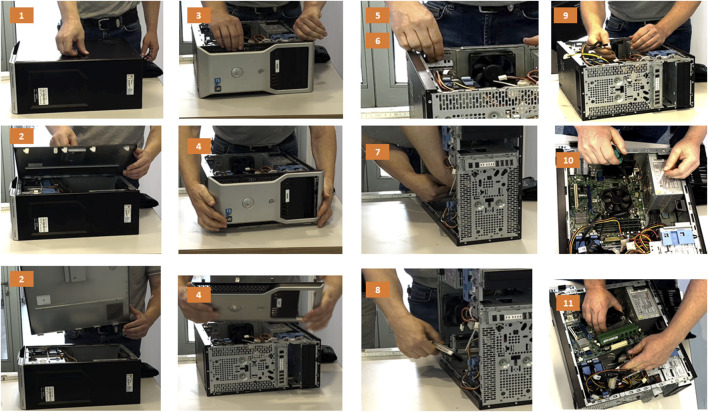
Photo documentation of the individual steps executed and documented in Supplementary Material.

In a next step, these tasks were executed and each individual process step was noted. In particular, the connections were listed as a combination of two components to be separated. The manual disassembly process that was then executed is documented (Figure 7 and Supplementary Material). Finally, the tools used for each step are documented. Again, the goal is to use the informal data models to determine whether the language is descriptive enough and contains the information relevant for the execution of the process.

These steps were repeated for all five of the computers used in the benchmarking exercise. The tables with the documentation of the disassembly processes using the informal data models are available in the data section.

## 3 Results and discussion

In this section, we will briefly present the results of the benchmarking exercise whereby the proposed methodology and informal data models were applied to the task of removing the mainboard from five computers that have reached their end-of-life. The results are divided into two sub-sections. First, we report on the manual disassembly process of removing the mainboard from the computer and highlight any additions or changes that were made to the informal data models we described in the previous section. Then we report on the work to translate the disassembly sequence into a robotic skill sequence.

For four of the five computers, user manuals describing maintenance were available online. These manuals included information on opening the housing were invaluable towards efficient disassembly. For one of the computers, no prior information was available. This information was extremely helpful, especially in determining the order of disassembly tasks. As an example, in the case of the computer Dell Precision T3500 from the year 2010, it was not immediately clear how to proceed after removing the initial cover. It was helpful to read that we should start with the hard drive carrier before continuing to the memory module housing. While we did not record the time required for the individual disassembly tasks or for each computer, it was definitely shorter for the computers with available manuals than for the other computer.

### 3.1 Results regarding informal data models

In the benchmarking exercise, five different computers were manually disassembled to extract the mainboard. The tables available in the Supplementary Material show the individual steps we executed to complete this goal for each of the computers. We used the language from the informal data models to describe the connections, the disassembly processes, and the tools that we would use for an automated disassembly of the same process (Table 5). In doing so, we identified that some of the connection types we encountered were not classifiable according to DIN 8593 and included connections that were either novel (such as cable ties) or that consist of a combination of other types of connection types (e.g., plugs which are inserted and which feature positive hooks for removal). These combinatorial connection types are furthermore associated with combinatory disassembly processes such as the need to press a latch and pull (for a cable connector), or in the case of cutting wires, to hold the wires with one hand while cutting using a tool in the other. In terms of the description of the condition of connections, we did not encounter a situation which challenged our initial model.

**TABLE 5:** Transposition from manual process to automated disassembly process using data models for PC #5 (Dell Precision T1600).

Corresponding step number from Table 4	Connection type A → B	Description of the connection	Disassembly process	Robot tool	Parameters of the robot tool
1	HINGE	spring-loaded, opening angle	Hold and move	Gripper	Suction pad D50
1	INSERTION	Swivel angle, removal direction	Hold and move	Gripper	Jaw gripper stroke 30 mm
2	CLAMPING	Contact direction and pressure force	Hold and move	Gripper	Jaw gripper stroke 10 mm
2	CLAMPING	Contact direction and pressure force	Hold and move	Gripper	Suction pad D100
3	HOOK	Direction and length of pressure	Hold and move	Gripper	Contact element 10 × 50
3	HINGE	Swivel axis and angle	Hold and move	Gripper	Jaw gripper stroke 30 mm
3	HOOK	Direction and length of pressure	Hold and move	Gripper	Contact element 10 × 50
3	POSITIVE LOCKING CONNECTORS	Plugs: PCI slot on mainboard	Hold and move	Gripper	Jaw gripper stroke 30 mm
4	POSITIVE LOCKING CONNECTORS	Plugs (some are very tight/stuck)	Holding and moving cutting	Gripper + side cutter	Jaw gripper stroke 10 mm cutting edge 20mm, D5mm
5	SCREWING	8 pieces, size PH2 (partly covered by cable)	Unscrew hold and move	Screwdriver Gripper	Bit PH2 jaw gripper stroke 10 mm
5	INSERTION	disturbing cables in the way, connections partly protrude into the rear panel	Holding and moving cutting	Gripper	Jaw gripper stroke 30 mm

### 3.2 Translation from disassembly sequence into skill-sequence

In this section, we discuss the conversion of the automatic disassembly plan into a skill-based robot program and some of challenges involved. The one-to-one mapping of executable skills to formal steps in the disassembly plan is not always possible. Our goal was to convert the defined, strongly linear sequence plan into a chain of skill invocations that is as linear as possible. Note, that this was still done manually in this experiment.

In some cases, additional steps are necessary for a feasible automation process. This includes preparing or post-processing in some of the respective steps. We have also mapped additional elements such as loops to special skill-blocks. They are used for later combinatorial optimization, especially of repetitive processes such as loosening screws that are distributed over different parts of the product. There could also be branches in the skill sequence, but this was not necessary in this example.

Table 6 lists all the skills identified as necessary for the disassembly example. These are also classified according to their type and briefly described in terms of the implemented ability.

**TABLE 6:** Skills used in exemplary disassembly process and classification according to complexity criteria.

Skill name	Type	Description of ability	LoHS	ToTR	InDp	InSt	LoC	CR	Total
reposition Object	Perception/Manipulation	manipulate whole object by putting it into a suitable orientation based on estimate of initial pose	1	0–1	1	0	0–1	1–2	3–6
Locate Object	Perception	locate object and identify verbal landmarks	2	-	0	0–1	1	0	3–4
Actuate Mechanism	Manipulation	manipulate various types of simple mechanisms using force guidance	0–1	0–2	0–1	0–2	1–2	0–1	1–9
Pick and Remove	Manipulation	hybrid action of picking up an object whereby said object needs to be dislodged by operating a simple mechanism using force guidance	0–1	1–2	0–1	0–1	1–2	0–1	2–8
Place Bin	Manipulation	drop an object into a bin	1–2	1	0	0	0–1	0–1	2–5
Bend and Break	Perception/Manipulation	permanently bend or even break latches or similar features by use of prying tools	0	1–2	0–1	0–1	1–2	0	2–6
Count Objects	Perception	detect, count, and locate the number of objects of a given type near the designated location (may include parameter of initial guesses)	2	-	0	0–1	1	0	3–4
Repeat for All	Logic	control logic modelled as skill block and includes combinatorial optimization strategies (using dynamically generated list or fixed parameter)	-	-	-	-	-	-	-
Cut Cables at Connector	Perception/Manipulation/AI	identify connector/socket of given type near the designated location and cut cable close to the connector	1	1–2	0–1	2	1–2	1	6–9
Uncrew	Perception/Manipulation	remove threaded fastener of designated type near designated location	1	1–2	0–1	0–1	1–2	1–2	4–9
Cleanup Workpiece	Manipulation	clean up by removing loose parts like screws from the workpiece	1	0–2	0–2	0–2	1	0–1	2–9
Pick and Remove (advanced)	Manipulation/AI	advanced version of the similar skill used to solve more complicated removal operations	0–1	1–2	0–1	2	1–2	1–2	5–10

The sequence of the skill invocations for the realization of the concrete process can be seen in Table 7. It becomes clear that some support skills were added during the conversion and, on the other hand, individual process steps had to be substituted according to the planned capabilities of the robot system. This applies to step 4.9, where it is too time-consuming to replicate the required dexterity with the robot in order to non-destructively release the different designs of latching plug connections. This step was substituted by a skill for cutting the cables at the connectors in the attached state.

**TABLE 7:** Overview of skills which would be used for mainboard extraction from a computer.

Step	Skill
1.1	Pre-position Object
1.1	Locate Object
1.1	Actuate Mechanism
1.2	Pick and Remove
1.2	Place Bin
2.3	Bend and Break
2.4	Pick and Remove
2.4	Place Bin
3.5	Bend and Break
3.6	Actuate Mechanism
3.7–3.8	Count Objects
3.7–3.8	Repeat for All
3.7	Actuate Mechanism
3.8	Pick and Remove
3.8	Place Bin
4.9	Repeat for All
4.9	Cut Cables at Connector
5.10	Count Objects
5.10	Unscrew
5.10	Clean-up Workpiece
5.11	Pick and Remove (advanced)
5.11	Place Bin

A particular challenge in the execution of our skill-based robot program is the spatial reference to component, sub-parts or particular features of the product or device. Especially for worn or defective devices to be disassembled, it is not guaranteed that exact coordinates of the location of features can be specified. For example, the device may be slightly deformed compared to the original CAD design model. To solve the problem, we have found it beneficial to add the possibility of entering verbal spatial descriptions as parameters for the “skills”. These descriptions can be defined manually but can also be derived automatically from more detailed model data. They are a simple form of a topological position description. They are simplified in the way that only a two-step localization is provided here. The first step is always the naming of the side of the object (top, bottom, right side). This is optionally followed by the localization of the area (back side, top right side). Alternatively, a feature arrangement can also be named here, for example, “the third screw from the top on the right side”.

**FIGURE 8:**
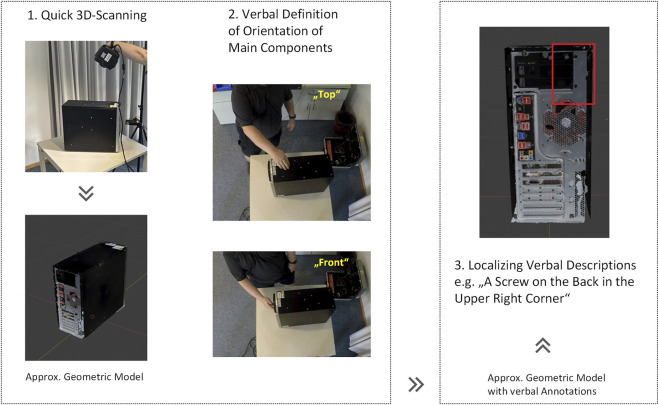
Modelling and localization process using verbal location information.

Figure 8 shows the process of interactive definition of our verbal coordinate system during experimentation. It starts with a rough 3D scan of the device. This can be made, for example, with the help of a handheld scanner or even with a smartphone. In a second step, the user defines the side views by pointing gestures and verbal utterances. This methodology represents an easy-to-use means for creating a sufficient geometric model for skill-based disassembly quickly and easily in the event that higher-quality model data is not available.

While planning our implementation, we have identified several attributes of skills that are relevant to identifying further requirements for the run-time system software as well as robot hardware, but can also be used to estimate or classify their respective complexity as a means for guiding design decisions during skill implementation. For this we used a point system whereby each aspect gets a score range based on individual criteria and contributes to an overall sum. Based on the author’s experience, the following attributes have been initially rated for the exemplary skills in Table 6.

• The Level of Hardware Specificity (LoHS) relates to the capability of the skill implementation to adapt to different robots. More abstraction from the specific robot hardware means generally more complex software implementation is needed. A score of 0 is assigned if only a specific type model is accepted, 1 means a rage of similar hardware is expected, and 2 would try to use anything that can be expressed by the model description format.
• Related to hardware is also the Type of Tool(s) required (ToTR). Here a score of 0 means no or simple passive tools are used, 1 represents off-the-shelf tools that can be commanded and may include limited sensors, while 2 is assigned if complex, skill-specific tools need to be developed and integrated.
• With the Interdependency (InDp) aspect we classify the reach of the skill across multiple assets. We assign a score of 0 if only a single asset without interdependencies needs to be commanded, 1 implies the coordination of multiple assets (e.g., robots together with a tool), and 2 would indicate an even higher degree of interdependency (e.g., collaboration with a human operator is also necessary).
• The aspect of Internal Statefulness (InSt) differentiates between the levels of context used during execution. If a skill can be used without parameters and is not dependent on any internal state across activations it is rated to be a 0. A score of 1 denote a skill that was concretized using manually provided information, manual tuning or training activities by the end-user. Skills with a mutable state that need to persist between activations get a 2. For the sake of keeping this classification simple, we include the criterion of polymorphism/subtyping of a skill into InSt: Any skill that can be used to drive sub-types needs to spawn instances with persistent internal state pertaining to the base-class (score >0).
• The Level of Containment (LoC) describes the vertical scope of a skill within a robot control architecture. A skill with a score of 0 can be executed entirely at the level of the main sequence executive. This includes skills whose implementation can also easily be containerized. A score of 1 denotes dependencies to specific, co-developed, high-level services. However, the most complex skills (score 2) require the development of specific real-time modules in order to implement the desired behavior.
• Required Compute Resources (CR) classify skills according to the time it takes to perform the required computation and thus the skill-execution model. A score of 0 means the skill is fully capable of live execution and updates DTs once after it finishes. Continuous updates of DTs during skill execution leads to a score of 1. If separate pre-processing steps like pre-planning, optimization, or post-processing phases are necessary outside the main execution context, the skill is considered more computationally complex and gets a score of 2.

A classification of skills according to the discussed criteria provided us with estimates of the complexity of their implementations. To make skill implementations manageable, those with particularly high overall scores could be candidates to be split up into multiple smaller ones that would need to be executes consecutively. The overall score could be used to prioritize which skills to initially focus on (e.g., starting with less-complex skills). Thus, according to our estimates, it would be advantageous to begin work with the skills “Locate Object, Count Objects”, and “Place Bin”.

## 4 Conclusion

This paper deals with the topic of automated disassembly for Re-X processes. The main contribution of this paper is our overall concept and methodology to use robots for the automated disassembly tasks for Re-X applications, combined with our suggestions for the role of data and the associated modeling of product and process data specific to the disassembly tasks. Building on the skills-based approach to robot programming from earlier research, we focus on the conceptual design of such skills that connects with the various levels of *a-priori* product data (e.g., complete, partial/incomplete, or completely missing product information). The desired performance of new robotic systems with respect to flexibility and speed, coupled with increasing levels of digitalization within the factory make the concept of skills-based robotic programming methods more immediate. There is a strong need for robot systems that can be programmed by a heterogeneous set of stakeholders with various backgrounds and levels of training with respect to robot programming and automation technology. By systematically considering the various sub-tasks involved in programming a robotics application, we formulated a set of requirements on a future-oriented, skills-based programming system. We then introduced a benchmarking scenario and evaluated existing methods/solutions for skills-based programming with particular focus on the requirements formulated in this work.

As an outlook we have a clear set of requirements that could be addressed by future robotics software developers and a workflow which also allows for other economic and sustainability criteria to be considered. Future work will focus on demonstrators for the various processes (identification, condition assessment, disassembly sequence determination, and automated disassembly) and their integration into a complete system for automated disassembly of WEEE which requires a minimum of engineering and programming efforts. This includes the engineering involved to specify the robotic hardware and peripheral components. Additionally, we expect to further refine and specify the informal models to support their integration into DTs.

## Data Availability

The original contributions presented in the study are included in the article/Supplementary Material, further inquiries can be directed to the corresponding author.
