# Supplementary material for: Automated disassembly of e-waste—requirements on modeling of processes and product states
Source: Front Robot AI. 2024 Mar 22;11:1303279. doi: 10.3389/frobt.2024.1303279 (PMC10995338; doi:10.3389/frobt.2024.1303279)
Supplement: Supplementary file 1 [file Table1.DOCX]

Supplementary material

TABLE 1: Manual Disassembly Process of PC #1 – Dell Precision T3400

| Single- stepS-No. | Connection Component A | Connection Component B | PROCESS DESCRIPTION | Used tool |
| --- | --- | --- | --- | --- |
| 1 | Release device | Computer | Push the release device backwards | Finger |
| 2 | Cover | Computer | Swing the cover Remove the cover | Finger |
| 3 | Release lever | Front panel | Pull the release lever of the front panel (pry up) | Finger |
| 4 | Front panel | Computer | Grab the front panel and pull it upwards | Finger |
| 5 | Release latch on card holder | Computer case | Press the latch and fold it open | Finger |
| 6 | Cover | Computer case | Press the release latch on the card retention cover and fold it open (pry up) | Finger |
| 7 | Lower card | Motherboard | Grab the card and loosen it by wiggling | Finger |
| 8 | Upper card | Computer case | Remove screw securing it to the case | Screwdriver and Finger |
| 9 | Cables | Computer case | Remove cables | Finger |
| 10 | Upper card | Motherboard | Press the PIC connection lever, grab the card and loosen and pull it by wiggling | Finger |
| 11 | Data cable (power supply) | System board | Disconnect the data cable (power supply) | Finger |
| 12 | Power cable | System board | Disconnect the power cable | Finger |
| 13 | Data cable (drives and hard disk) | System board | Disconnect the data cable (drives and hard disk) | Finger |
| 14 | Data cable (I/O) | System board | Disconnect the data cable (I/O) | Finger |
| 15 | Screws | System board | Unscrew the 12 screws | Screwdriver and Finger |
| 16 | System board | Computer | Lift the system board out of the computer | Finger |
| 17 | Front security screws on the heatsink | System board | Loosen the two front security screws | Screwdriver and Finger |
| 18 | Heatsink | System board | Tilt the heatsink at the hinge | Finger |
| 19 | Rear security screws on the heatsink | System board | Loosen the two rear security screws | Screwdriver and Finger |
| 20 | Heatsink | System board | Grab and remove the component for securing the heatsink | Finger |
| 21 | USB cable | System board | Remove cable | Finger |
| 22 | System board | Computer case | Grab the system board, lever it forward and pull it out | Finger |

TABLE 2: Manual Disassembly Process of PC #2 – Dell Precision T3500

| Single- stepS-No. | Connection Component A | Connection Component B | PROCESS DESCRIPTION | Used tool |
| --- | --- | --- | --- | --- |
| 1 | Release device | Computer | Push the release device backwards | Finger |
| 2 | Cover | Computer | Swing the cover Remove the cover | Finger |
| 3 | Release latch | Computer | Press down and hold the release latch | Finger |
| 4 | Hard drive carrier | Computer | Swing the hard drive carrier | Finger |
| 5 | Memory module housing | System board | Lift the memory module housing straight upwards | Finger |
| 6 | Fan cables | System board | Disconnect the fan cables from the system board | Finger |
| 7 | Screw | Computer | Unscrew the screw | Screwdriver and Finger |
| 8 | Fan assembly | Computer | Lift the fan assembly straight upwards | Finger |
| 9 | Bracket arm | Computer | Swing the bracket arm upwards | Finger |
| 10 | Locking latch (Bracket arm) | Bracket arm | Press and hold the locking latches (2-finger) | Finger |
| 11 | Bracket arm | Computer | Swing the bracket arm completely outward | Finger |
| 12 | USB cable | Expansion card | Disconnect the USB cable from the expansion card | Finger |
| 13 | Locking latch (Expansion card) | System board | Pull the blue locking latch (expansion card) away from the graphics card and hold it | Finger |
| 14 | Expansion card | System board | Pull the graphics card upwards | Finger |
| 15 | Data cable (power supply) | System board | Disconnect the data cable (power supply) | Finger |
| 16 | Power cable | System board | Disconnect the power cable | Finger |
| 17 | Data cable (drives and hard disk) | System board | Disconnect the data cable (drives and hard disk) | Finger |
| 18 | Data cable (I/O) | System board | Disconnect the data cable (I/O) | Finger |
| 19 | Screws | System board | Unscrew the 12 screws | Screwdriver and Finger |
| 20 | System board | Computer | Lift the system board out of the computer | Finger |

TABLE 3: Manual Disassembly Process of PC #3 – TAROX

| Single- stepS-No. | Connection Component A | Connection Component B | PROCESS DESCRIPTION | Used tool |
| --- | --- | --- | --- | --- |
| 1 | Release | Cover | Push the release device in backward direction | Finger |
| 2 | Cover | Computer | Remove the cover from the computer (move backwards and lift) | Finger |
| 3 | Screw | Computer | Unscrew the screw from the graphics card holder | Screwdriver and Finger |
| 4 | Graphics card | Computer | Pull the graphics card away from system board (high pulling force) | Finger |
| 5 | Screw | Computer | Unscrew the screw from the network card holder | Screwdriver and Finger |
| 6 | Wiring | Network card | Cut the wirings between network card and system board | Wire cutter |
| 7 | Network card | Computer | Pull the network card away from system board | Finger |
| 8 | 21 pin ATX connector | System board | Cut the ATX connector cable | Wire cutter |
| 9 | 12V connector | System board | Cut the power supply cable | Wire cutter |
| 10 | SATA connectors (3x) | System board | Cut the SATA cable | Wire cutter |
| 11 | Case fans | System board | Cut the fan cable | Wire cutter |
| 12 | Power supply | Computer | Pull out the power supply from the cable bundle | Finger |
| 13 | Screws PH2 | Back side | Unscrew the screws fixing the system board to the case | Screwdriver and Finger |
| 14 | System board | Computer | Lift the system board out of the case | Finger |

TABLE 4: Manual Disassembly Process of PC #4 and #5 – Dell Precision T1600

| Single- stepS-No. | Connection Component A | Connection Component B | PROCESS DESCRIPTION | Used tool |
| --- | --- | --- | --- | --- |
| 1 | Enabling device | Cover | Swiveling and holding the release device (handle) | Finger |
| 2 | Cover | Computer | Lifting the cover at an angle of 45° and removing it from the computer | Finger |
| 3 | Retaining clips | Computer | Pressing (levering away) the retaining clips from the computer | Finger |
| 4 | Front panel | Computer | Rotating the front panel away from the computer | Finger |
| 5 | Card retainer seal | Computer | Pressing the release tab on the card retaining latch | Finger |
| 6 | Card retainer seal | Computer | Swinging the card retainer latches outward | Finger |
| 7 | Unlocking lever | Computer | Pressing the unlocking lever | Finger |
| 8 | Expansion card | System board | Dragging the card upwards | Finger |
| 9 | Different cables | System board | Pulling the cables off the system board (pressing in hooks partly necessary) | Finger |
| 10 | Screws | System board | Unscrewing the screws and removing the screws | Screwdriver and Finger |
| 11 | System board | Computer | Sliding the system board to the front  Swiveling the system board by 45°  Lifting out the system board (no linear movement possible due to connections) | Finger |
